# Intensive Rehabilitation Treatment in Parkinsonian Patients with Dyskinesias: A Preliminary Study with 6-Month Followup

**DOI:** 10.1155/2012/910454

**Published:** 2012-06-03

**Authors:** Giuseppe Frazzitta, Micaela Morelli, Gabriella Bertotti, Guido Felicetti, Gianni Pezzoli, Roberto Maestri

**Affiliations:** ^1^Department of Neurorehabilitation, Scientific Institute of Montescano, S. Maugeri Foundation IRCCS, 27040 Montescano, Italy; ^2^Department of Toxicology and Centre of Excellence for Neurobiology of Dependence, University of Cagliari, and CNR Institute for Neuroscience, 09126 Cagliari, Italy; ^3^Parkinson Institute, Istituti Clinici di Perfezionamento, Milano, Italy; ^4^Department of Biomedical Engineering, Scientific Institute of Montescano, S. Maugeri Foundation IRCCS, 27040 Montescano, Italy

## Abstract

A major adverse effect of levodopa therapy is the development of dyskinesia, which affects 30–40% of chronically treated Parkinsonian patients. We hypothesized that our rehabilitation protocol might allow a reduction in levodopa dosage without worsening motor performances, thus reducing frequency and severity of dyskinesias. Ten Parkinsonian patients underwent a 4-week intensive rehabilitation treatment (IRT). Patients were evaluated at baseline, at the end of the rehabilitation treatment and at 6-month followup. Outcome measures were the Unified Parkinson's Disease Rating Scale Sections II, III, and IV (UPDRS II, III, IV) and the Abnormal Involuntary Movement Scale (AIMS). At the end of the IRT, levodopa dosage was significantly reduced (*P* = 0.0035), passing from 1016 ± 327 to 777 ± 333 mg/day. All outcome variables improved significantly (*P* < 0.0005 all) by the end of IRT. At followup, all variables still maintained better values with respect to admission (*P* < 0.02 all). In particular AIMS score improved passing from 11.90 ± 6.5 at admission to 3.10 ± 2.3 at discharge and to 4.20 ± 2.7 at followup. Our results suggest that it is possible to act on dyskinesias in Parkinsonian patients with properly designed rehabilitation protocols. Intensive rehabilitation treatment, whose acute beneficial effects are maintained over time, might be considered a valid noninvasive therapeutic support for Parkinsonian patients suffering from diskinesia, allowing a reduction in drugs dosage and related adverse effects.

## 1. Introduction

A variety of drugs have been developed in the last fifty years and are currently used to control the disability related to Parkinson's disease (PD): levodopa, dopamine-agonists, monoaminoxidase B inhibitors, catechol-*O*-methyltransferase inhibitors.

A major limiting factor in levodopa therapy is the development of motor complications, in particular dyskinesia, which affects 30–40% of chronically treated PD patients [1].

Dyskinesias can improve by reducing the dopaminergic therapy, but it is usually cumbersome to decrease the levodopa dosage since this reduction elicits a worsening of motor symptoms: an increased bradykinesia, an increased “off time,” a reduction of motor performance, and autonomy in daily activities.

In the last decade, a considerable number of studies have shown that exercise is effective in improving gait, balance, freezing, and motor performance in PD. In particular, recent studies on animals allow hypothesizing a direct action of physical activity on the mechanisms responsible for dyskinesias [2, 3].

In this study we present preliminary data on the effectiveness of intensive rehabilitation treatment (IRT) in PD patients with dyskinesias and on the persistence over time of its beneficial effects.

## 2. Methods

### 2.1. Study Population

Patients were screened from among those consecutively admitted to the movement disorder ambulatories of the Rehabilitation Institute of Montescano. Eligibility criteria for patients were (a) diagnosis of “clinically probable” idiopathic Parkinson's disease according to Gelb et al. [4], (b) development of dyskinesias in the last 3 years and a history of several failed attempts to improve dyskinesia by reducing or modifying drug dosage, (c) ability to walk without any physical assistance, (d) no cognitive impairment (mini-mental state examination score ≥26), (e) no comorbidity unrelated to Parkinson's disease, (f) no vestibular/visual dysfunction limiting locomotion or balance, and (h) antiparkinsonian medications stable for >4 weeks.

Ten eligible patients were invited to be admitted to the Rehabilitation Institute of Montescano for a 4-week intensive rehabilitation treatment.

Patients were examined by the same neurologist expert in movement disorders, in the morning, one hour after they had taken the first dose of levodopa, at baseline, at the end of the rehabilitation treatment, and at 6-month followup. The neurologist was blinded with respect to the study design for the entire period.

The outcome measures used were the Unified Parkinson's Disease Rating Scale Sections II, III, and IV (UPDRS II, III, IV) [5] and the Abnormal Involuntary Movement Scale (AIMS) [6].

Patients were treated with different drugs (levodopa, I-COMT, I-MAOB, or dopamine agonist), and we evaluated the drug dosage as levodopa equivalent (mg/day).

The study was approved by the local ethics committee, and all subjects gave their informed written consent before participation.

### 2.2. Intervention

IRT consisted of a 4-week cycle of physiotherapy that entailed three daily sessions (two, not consecutive, in the morning and one in the afternoon), 5 days a week. The global duration of each session, including recovery periods, was about one hour. The first session comprised cardiovascular warm-up activities, relaxation exercises, muscle-stretching exercises (scapular muscle group, hip flexor, hamstring and gastrocnemius muscles), exercises to improve the range of motion of spinal, pelvic, and scapular joints, exercises to improve the functionality of the abdominal muscles, and postural changes in the supine position.

The second session comprised exercises to improve balance and gait using a stabilometric platform with a visual cue (patients were asked to follow a circular pathway on the screen by using a cursor sensitive to their feet movements on the platform) and treadmill plus (treadmill training with both a visual and an auditory cue) [7]. The last session was a session of occupational therapy aimed at improving autonomy in daily living activities: transferring from sitting position to standing position, rolling from supine position to sitting position and from sitting to supine, dressing, use of tools, and exercises to improve hand functionality and skills (e.g., using screws and bolts). Moreover, patients spent 20 minutes every day in front of a mirror in order to control involuntary and exaggerated movements.

During the follow-up period, patients were invited to continue some simple exercises learnt during hospitalization period.

### 2.3. Statistical Analysis

Descriptive statistics are given as mean ± SD. The Shapiro-Wilk statistic was used to test the normality of the distribution of all variables.

The effect of treatment on each outcome variable and the persistence over the 6-month follow-up period were assessed by repeated measurements analysis of variance with three repeated measurements: admission, discharge, and 6-month followup. Pairwise comparisons (discharge versus admission and 6-month followup versus admission) were carried out by contrast analysis in repeated measurements analysis of variance. A *P* value <0.05 was considered statistically significant. All analyses were carried out using the SAS/STAT statistical package, release 9.2 (SAS Institute Inc., Cary, NC, USA).

## 3. Results

All 10 patients (aged  70 ± 8  years, duration of the disease 11.4 ± 2.4 years) completed the intensive rehabilitation treatment and the 6-month follow-up control. The characteristics of patients at admission, discharge and at the follow-up time are reported in Table 1.

At the end of IRT, levodopa-equivalent dosage was significantly reduced (*P* = 0.0035), passing from  1016 ± 327  to 777 ± 333 mg/day. At follow-up the levodopa-equivalent dosage was unchanged.

All outcome variables improved significantly by the end of the rehabilitation treatment (*P* = 0.0003, *P* < 0.0001, *P* < 0.0001 and *P* = 0.0005 for UPDRS_II, UPDRS_III, UPDRS_IV and AIMS, resp.). At followup, all variables still maintained better values with respect to admission (*P* = 0.0176, *P* < 0.0001, *P* < 0.0001 and *P* = 0.0026, resp.).

## 4. Conclusion

In this study we investigated the efficacy of IRT in PD patients with dyskinesias and the persistence over time of the beneficial effects of this treatment. We found a statistically and clinically significant improvement in all outcome variables after the 4-week rehabilitation period, which was largely preserved even after a 6-month period.

The improvement in UPDRS II and III observed in this study is in accordance with our preview studies, in which we demonstrated that IRT acts slowing the disease progression in Parkinsonian patients in a very long followup [8]. The patients continued to perform the recommended exercises during the follow-up period and this may explain the persistence of the beneficial effects obtained during hospitalization. Moreover, the simple reduction of intensity and duration of dyskinesias during the day leads the patients to improve their motor performance and autonomy during activity of daily life.

 Our results suggest that it is possible to act on dyskinesias in Parkinsonian patients with an IRT. Several preclinical investigations carried out in animal models of PD have demonstrated that an overload of redundant motor information is stored in the basal ganglia motor circuits of dopamine-denervated animals.

In particular, the striatum receives the most important glutamatergic innervation, is the site of interaction glutamate/dopamine, is the source of the inhibitory outputs, and is involved in the generation of motor fluctuation linked to L-dopa treatment [2]. In animal models, after denervation, the striatal plasticity is lost, but the chronic L-dopa treatment is able to restore the long term potentiation (LTP) of synaptic transmission [9, 10].

The reversal of synaptic strength from the potentiated state to pre-LTP levels is named depotentiation, and this process represents the synaptic process of erasing unnecessary motor information. In Parkinsonian animal models treated with L-dopa which show dyskinesias movement, the synaptic depotentiation is lost [2]. The inability of corticostriatal synapses to depotentiate might represent the cellular basis of dyskinesias.

The execution of movements plays a fundamental role in determining the outcome of subsequent motor responses elicited by dopamine receptor stimulation [11]. Exaggerated movements in response to a stimulation of dopaminergic receptors, such as those occurring during dyskinesia, might consequently convey erroneous information to the motor striate circuits. Therefore, when concomitant, competing correct movements are performed (as during rehabilitation treatment), the manifestation of abnormal dyskinetic movements may be attenuated.

This study, therefore, suggests the possibility that the competition between a correct motor behaviour and an abnormal motor response may depend on the balance between the trace memory of the two.

Another possible explanation may be related to a neurorestorative strategy. The effects of intensive exercise in promoting cell proliferation and neuronal differentiation in animal models are reported in a large cohort of studies.

In animals with cerebral lesions produced by 6-hydroxydopamine (6-OHDA) or 1-methyl-4-phenyl-1,2,3,6-tetrahydropyridine (MPTP), the intensive use of a treadmill or running wheels led to improvement in motor performance as compared to animals that did not use these devices. Both in unilateral and bilateral models of PD, intensive treadmill exercise produced improvement in motor symptoms, which was related to a reduction in the neurochemical deficit: preservation of both tyrosine hydroxylase-positive fibres in the striatum and substantia nigra, as well as of vesicular monoamine transporter and dopamine transporter levels [12–17]. Increased dopamine availability, especially within the dorsolateral striatum, has been found in an MPTP mice model after intensive exercise with a motorized treadmill [18]. Overall, these findings show that intensive exercise exerts beneficial effects on dopamine transmission in parkinsonian mice models.

These neuroplastic effects of intensive exercise are probably related to increased expression of a variety of neurotrophic factors. In particular, brain-derived neurotrophic factor (BDNF) and glia-derived neurotrophic factor (GDNF) are the most likely growth factors involved in this process. BDNF is a key component of a number of aspects of neuroplasticity: neurogenesis, synaptogenesis, and cell survival [19, 20], while GDNF has been shown to promote the survival and differentiation of dopamine neurons and to maintain the survival of adult catecholaminergic neurons in mice [21, 22]. Tajiri et al. [17] have recently shown that rat models of PD performing intensive treadmill exercise experience upregulation of BDNF and GDNF in the striatum in comparison to rats that do not exercise. These findings are consistent with the findings of another study by Lau et al. [23], who showed that intensive treadmill exercise raises the level of endogenous BDNF and GDNF in the substantia nigra and striatum.

In conclusion, our findings suggest that properly designed intensive multidisciplinary rehabilitation treatment using treadmill should be considered as a valid noninvasive therapeutic support for patients who show dyskinesias.

## Figures and Tables

**Table 1:** Outcome variables at admission, at discharge after a 4-week intensive rehabilitation treatment, and at 6-month followup.

Variable	Admission	Discharge	6-month
			followup
UPDRS_II	14.30 ± 4.7	9.40 ± 5.1	9.40 ± 3.0
UPDRS_III	20.00 ± 4.9	14.10 ± 4.3	11.60 ± 4.1
UPDRS_IV	7.50 ± 3.7	1.70 ± 1.6	2.60 ± 2.1
AIMS	11.90 ± 6.5	3.10 ± 2.3	4.20 ± 2.7
Levodopa equivalent	1016 ± 327	777 ± 333	777 ± 333
(mg/day)			

UPDRS: Unified Parkinson's Disease Rating Scale (Sections II, III, and IV).

AIMS: Abnormal Involuntary Movement Scale.

## References

[1] Jankovic J (2005). Motor fluctuations and dyskinesias in Parkinson’s disease: clinical manifestations. *Movement Disorders*.

[2] Picconi B, Centonze D, Håkansson K (2003). Loss of bidirectional striatal synaptic plasticity in L-DOPA-induced dyskinesia. *Nature Neuroscience*.

[3] Pisani A, Centonze D, Bernardi G, Calabresi P (2005). Striatal synaptic plasticity: implications for motor learning and Parkinson’s disease. *Movement Disorders*.

[4] Gelb DJ, Oliver E, Gilman S (1999). Diagnostic criteria for Parkinson disease. *Archives of Neurology*.

[5] Fahn S, Elton RL, Fahn S, Marsden CD, Calne D, Goldstein M (1987). Unified Parkinson’s disease rating scale. *Recent Developments in Parkinson’s Disease*.

[6] Simpson GM, Lee JH, Zoubok B, Gardos G (1979). A rating scale for tardive dyskinesia. *Psychopharmacology*.

[7] Frazzitta G, Maestri R, Uccellini D, Bertotti G, Abelli P (2009). Rehabilitation treatment of gait in patients with Parkinson’s disease with freezing: a comparison between two physical therapy protocols using visual and auditory cues with or without treadmill training. *Movement Disorders*.

[8] Frazzitta G, Bertotti G, Riboldazzi G (2012). Effectiveness of intensive inpatient rehabilitation treatment on disease progression in Parkinsonian patients: a randomized controlled trial with 1-year follow-up. *Neurorehabil Neural Repair*.

[9] Centonze D, Picconi B, Gubellini P, Bernardi G, Calabresi P (2001). Dopaminergic control of synaptic plasticity in the dorsal striatum. *European Journal of Neuroscience*.

[10] Centonze D, Gubellini P, Picconi B, Calabresi P, Giacomini P, Bernardi G (1999). Unilateral dopamine denervation blocks corticostriatal LTP. *Journal of Neurophysiology*.

[11] Simola N, Di Chiara G, Daniels WMU, Schallert T, Morelli M (2009). Priming of rotational behavior by a dopamine receptor agonist in hemiparkinsonian rats: movement-dependent induction. *Neuroscience*.

[12] Freed CR, Yamamoto BK (1985). Regional brain dopamine metabolism: a marker for the speed, direction, and posture of moving animals. *Science*.

[13] MacRae PG, Spirduso WW, Cartee GD, Farrar RP, Wilcox RE (1987). Endurance training effects on striatal D2 dopamine receptor binding and striatal dopamine metabolite levels. *Neuroscience Letters*.

[14] Liste I, Guerra MJ, Caruncho HJ, Labandeira-Garcia JL (1997). Treadmill running induces striatal Fos expression via NMDA glutamate and dopamine receptors. *Experimental Brain Research*.

[15] Wang GJ, Volkow ND, Fowler JS (2000). PET studies of the effects of aerobic exercise on human striatal dopamine release. *Journal of Nuclear Medicine*.

[16] Tillerson JL, Caudle WM, Reverón ME, Miller GW (2003). Exercise induces behavioral recovery and attenuates neurochemical deficits in rodent models of Parkinson’s disease. *Neuroscience*.

[17] Tajiri N, Yasuhara T, Shingo T (2010). Exercise exerts neuroprotective effects on Parkinson’s disease model of rats. *Brain Research*.

[18] Petzinger GM, Fisher BE, Van Leeuwen JE (2010). Enhancing neuroplasticity in the basal ganglia: the role of exercise in Parkinson’s disease. *Movement Disorders*.

[19] Cotman CW, Berchtold NC, Christie LA (2007). Exercise builds brain health: key roles of growth factor cascades and inflammation. *Trends in Neurosciences*.

[20] Mattson MP, Maudsley S, Martin B (2004). A neural signaling triumvirate that influences ageing and age-related disease: insulin/IGF-1, BDNF and serotonin. *Ageing Research Reviews*.

[21] Lin LF, Doherty DH, Lile JD, Bektesh S, Collins F (1993). GDNF: a glial cell line-derived neurotrophic factor for midbrain dopaminergic neurons. *Science*.

[22] Pascual A, Hidalgo-Figueroa M, Piruat JI, Pintado CO, Gómez-Díaz R, López-Barneo J (2008). Absolute requirement of GDNF for adult catecholaminergic neuron survival. *Nature Neuroscience*.

[23] Lau YS, Patki G, Das-Panja K, Le WD, Ahmad SO (2011). Neuroprotective effects and mechanisms of exercise in a chronic mouse model of Parkinson’s disease with moderate neurodegeneration. *European Journal of Neuroscience*.

